# Integrating multi-omics analyses of *Nonomuraea dietziae* to reveal the role of soybean oil in [(4′-OH)MeLeu]^4^-CsA overproduction

**DOI:** 10.1186/s12934-017-0739-0

**Published:** 2017-07-14

**Authors:** Huanhuan Liu, Di Huang, Lina Jin, Cheng Wang, Shaoxiong Liang, Jianping Wen

**Affiliations:** 1 Key Laboratory of System Bioengineering (Tianjin University), Ministry of Education, Tianjin, 300072 People’s Republic of China; 2 SynBio Research Platform, Collaborative Innovation Center of Chemical Science and Engineering (Tianjin), School of Chemical Engineering and Technology, Tianjin University, Tianjin, 300072 People’s Republic of China; 3 TEDA Institute of Biological Sciences and Biotechnology, Nankai University, TEDA, Tianjin, 300457 People’s Republic of China; 4 SynBio Research Platform, Collaborative Innovation Center of Chemical Science and Engineering (Tianjin), Nankai University, Tianjin, 300071 People’s Republic of China

**Keywords:** [(4′-OH)MeLeu]^4^-CsA, *Nonomuraea dietziae*, Soybean oil, Proteomics, Metabolomics, Cytochrome P450 hydroxylases

## Abstract

**Background:**

*Nonomuraea dietziae* is a promising microorganism to mediate the region-specific monooxygenation reaction of cyclosporine A (CsA). The main product [(4′-OH)MeLeu]^4^-CsA possesses high anti-HIV/HCV and hair growth-stimulating activities while avoiding the immunosuppressive effect of CsA. However, the low conversion efficiency restricts the clinical application. In this study, the production of [(4′-OH)MeLeu]^4^-CsA was greatly improved by 55.6% from 182.8 to 284.4 mg/L when supplementing soybean oil into the production medium, which represented the highest production of [(4′-OH)MeLeu]^4^-CsA so far.

**Results:**

To investigate the effect of soybean oil on CsA conversion, some other plant oils (corn oil and peanut oil) and the major hydrolysates of soybean oil were fed into the production medium, respectively. The results demonstrated that the plant oils, rather than the hydrolysates, could significantly improve the [(4′-OH)MeLeu]^4^-CsA production, suggesting that soybean oil might not play its role in the lipid metabolic pathway. To further unveil the mechanism of [(4′-OH)MeLeu]^4^-CsA overproduction under the soybean oil condition, a proteomic analysis based on the two-dimensional gel electrophoresis coupled with MALDI TOF/TOF mass spectrometry was implemented. The results showed that central carbon metabolism, genetic information processing and energy metabolism were significantly up-regulated under the soybean oil condition. Moreover, the gas chromatography-mass spectrometry-based metabolomic analysis indicated that soybean oil had a great effect on amino acid metabolism and tricarboxylic acid cycle. In addition, the transcription levels of cytochrome P450 hydroxylase (CYP) genes for CsA conversion were determined by RT-qPCR and the results showed that most of the CYP genes were up-regulated under the soybean oil condition.

**Conclusions:**

These findings indicate that soybean oil could strengthen the primary metabolism and the CYP system to enhance the mycelium growth and the monooxygenation reaction, respectively, and it will be a guidance for the further metabolic engineering of this strain.

**Electronic supplementary material:**

The online version of this article (doi:10.1186/s12934-017-0739-0) contains supplementary material, which is available to authorized users.

## Background

Cyclosporine A (CsA), produced by soil fungus *Tolypocladium niveum*, is a natural cyclic undecapeptide possessing immunosuppressive activity, and is the active ingredient of Sandimmune^®^ and Neoral^®^ for preventing organ transplant rejection [1]. Besides, CsA is reported to substantially inhibit the virus replication of HCV (hepatitis C virus) [2, 3] and HIV-1 (human immunodeficiency virus type 1) [4]. However, developing a CsA-based anti-HIV/HCV drug should exclude the immunosuppressive activity of CsA since it will antagonize the host immune system for clearing virus [5]. Fortunately, this problem has been effectively solved by modifying the side chain of the [MeLeu]^4^ residue [6]. Among these derivatives, [(4′-OH)MeLeu]^4^-CsA has a high anti-HIV/HCV activity while significantly lowering the immunosuppressive activity [6, 7]. Meanwhile, [(4′-OH)MeLeu]^4^-CsA is a starting point to trigger the search for other promising analogues, e.g., [(D)MeSer]^3^-[(4′-OH)MeLeu]^4^-CsA and [Sar-D-OMe]^3^[(4-OH)MeLeu]^4^-CsA, both of whose anti-HIV activities are more than sevenfold of CsA [8, 9]. Recently, [(4′-OH)MeLeu]^4^-CsA has also been reported to possess the hair growth-promoting effect [7, 10], which attracted much attention from both academy and cosmetics industry due to its potential value to treat alopecia [11, 12].

*Nonomuraea dietziae* (*Sebekia benihana*), a rare soil actinomycete, is one of the best microorganisms that can efficiently convert CsA to the region-specific hydroxylation product [(4′-OH)MeLeu]^4^-CsA [13] by cytochrome P450 hydroxylases (CYPs) [14]. To improve the bioconversion rate of CsA, various strategies have been applied so far, such as genetic manipulation [14, 15], medium optimization and traditional mutation. Lee et al. overexpressed CYP-sb21 gene in wild-type *N. dietziae* and increased the conversion rate of CsA by twofold (reaching 29%) [16]. By the same token, a 54% conversion rate was obtained when molybdenum salt was added into the optimized medium [17]. However, the product titers and yields of these strains are still at a low level, and impede the industrial application of [(4′-OH)MeLeu]^4^-CsA.

Recently, we have successfully obtained a mutant strain with the high [(4′-OH)MeLeu]^4^-CsA production by UV-LiCl complex mutation. Interestingly, addition of 0.1% (w/v) soybean oil could further increase the conversion rate by 55.6%. Although soybean oil has been proved to accelerate the strain’s growth and improve the antibiotic production [18–20], the specific effects on [(4′-OH)MeLeu]^4^-CsA production may be complex and comprehensive because the conversion process from CsA to [(4′-OH)MeLeu]^4^-CsA is a monooxygenation reaction and seems irrelevant to soybean oil. Hence, to unveil the potential mechanism, in this study, the two-dimensional gel electrophoresis (2-DE) coupled with matrix-assisted laser-desorption/ionization time-of-flight/time-of-flight mass spectrometry (MALDI-TOF/TOF–MS) and gas chromatography–mass spectrometry (GC–MS), were employed for proteomic and metabolomic analyses, respectively. Moreover, the transcription levels of CYP genes were analyzed by quantitative real-time PCR (qRT-PCR) to investigate the role of the soybean oil on the overproduction of [(4′-OH)MeLeu]^4^-CsA.

## Results and discussion

### Effects of the exogenous soybean oil on the fermentation properties of *N. dietziae*

**Fig. 1:**
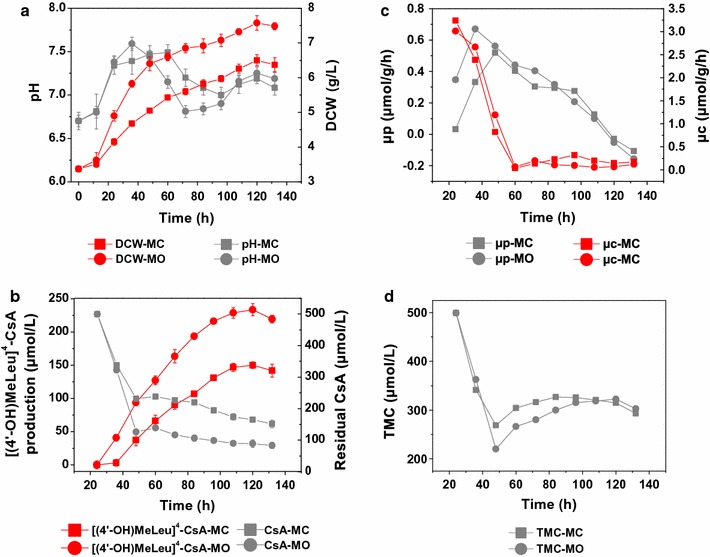
Time course profiles of key fermentation parameters in 500 mL shaking flask. **a** Dynamic fermentation profiles of dry cell weight and pH; **b** Concentration of [(4′-OH)MeLeu]^4^-CsA and CsA in medium MO and MC; **c** Changes of the specific rates in medium MO and MC; **d** The total mole concentration of CsA and [(4′-OH)MeLeu]^4^-CsA. Each value represents the mean of five independent experiments and the *error bars* represent standard deviations of five values. *MO* soybean oil medium, *MC* soybean oil-free medium; *μ*_*p*_, specific production rate of [(4′-OH)MeLeu]^4^-CsA, *μ*_*c*_ specific consumption rate of CsA, *TMC* total mole concentration of CsA and [(4′-OH)MeLeu]^4^-CsA

As shown in Fig. 1, the major fermentation features of *N. dietziae* in soybean oil medium (assigned as the medium MO) were distinctly different from the control group (medium MC). The initial 12 h was the lag phase and the both biomass changed little, while the following log phase (12–48 h) displayed significant discrepancies (Fig. 1a). During the log phase, biomass in the medium MO increased sharply from 3.63 to 6.4 g/L (improved by 76.3%, Fig. 1a), compared with a relatively gentle change in the medium MC (improved by 43.5%, Fig. 1a), indicating a more appropriate growth environment under the MO condition. Throughout the whole fermentation process, the highest biomass (7.58 g/L) and [(4′-OH)MeLeu]^4^-CsA titer (233.4 μmol/L or 284.4 mg/L) were achieved at 120 h in the medium MO, 1.16- and 1.56-folds compared to that in the medium MC, respectively (Fig. 1a, b). After 120 h, the [(4′-OH)MeLeu]^4^-CsA production declined gradually due to the product degradation.

In this study, the intrinsic conversion capacity of *N. dietziae* per unit of biomass was characterized by the specific production rate (μ_p_), an important kinetic parameter that removed the impact of biomass on the [(4′-OH)MeLeu]^4^-CsA production. As shown in Fig. 1c, the maximum μ_p_ was achieved in the medium MO (0.67 μmol/g/h, 36 h), which was 1.3-folds and appeared 12 h earlier than that in the MC medium (0.51 μmol g/h, 48 h). Although the μ_p_ of MO was lower than MC during the late fermentation period (from 84 h to 120 h, Fig. 1c), yet the most production of [(4′-OH)MeLeu]^4^-CsA achieved at 84 h in both media (Fig. 1b), indicating that the catalytic capacity of medium MO was superior to MC.

In addition, the concept “total mole concentration” (TMC) was put forward to describe the total mole concentration of the residual CsA and the produced [(4′-OH)MeLeu]^4^-CsA in the fermentation broth media based on the fact that the hydroxylation process is a monooxygenation reaction, in which the conversion coefficient of CsA to [(4′-OH)MeLeu]^4^-CsA is 1 mol:1 mol. In Fig. 1d, the TMC reached the minimum value at 48 h, but significantly increased during the late fermentation period in both medium. This phenomenon indicated that part of CsA was probably converted into other intermediates, such as the CsA-CYPs complex, as mentioned previously [16, 21]. Accordingly, the lower value of TMC in the MO medium represented a stronger intermediate processing capacity (or catalytic ability) than that in the control.

### Effects of the major hydrolysates of soybean oil and some other plant oils (corn oil and peanut oil) on [(4′-OH)MeLeu]^4^-CsA production

Since soybean oil is a natural lipid mixture and can be hydrolyzed into fatty acids and glycerol, both of which will subsequently participate in the metabolic system [19, 22]. Considering the potential association between the lipid metabolism and the soybean oil, series of feeding experiments were designed and carried out, including the main hydrolysates of soybean oil, such as oleic acid, linoleic acid and glycerol, as well as the soybean oil-like compounds, such as corn oil (0.1%, w/v), peanut oil (0.1%, w/v) and Tween 80 (polyoxyethylene sorbitan monooleate, 0.05%, w/v). All the compounds were fed into the medium MC at the beginning of fermentation.

**Fig. 2:**
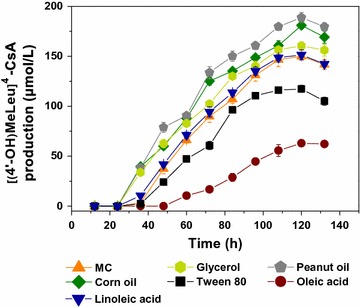
Effects of major hydrolysates of soybean oil and some other plant oils (corn oil and peanut oil) on [(4′-OH)MeLeu]^4^-CsA production. Each value represents the mean of five independent experiments and the *error bars* represent standard deviations of five values

As shown in Fig. 2, oleic acid, linoleic acid and Tween 80 all failed to enhance the conversion of CsA to [(4′-OH)MeLeu]^4^-CsA. Glycerol was an exception since the [(4′-OH)MeLeu]^4^-CsA production had a 7% increase. Considering that glycerol was probably used as a complementary carbon source, and that increasing the concentration of glycerol could not further enhance the CsA conversion (data not shown), a 7% increase, nevertheless, was still much smaller compared with the impact of soybean oil (55.6%). Thus, it could be concluded that the main hydrolysates of soybean oil contributed little or even negatively to the improvement of CsA conversion.

Addition of corn oil and peanut oil, by contrast, led to the 20.7 and 25.8% increases in [(4′-OH)MeLeu]^4^-CsA production, respectively, suggesting that plant oils (soybean oil, corn oil and peanut oil) could efficiently improve the conversion of CsA. Therefore, although *N. dietziae* cannot directly utilize the hydrolysates of plant oil to strengthen mycelium growth and [(4′-OH)MeLeu]^4^-CsA synthesis, the overproduction of [(4′-OH)MeLeu]^4^-CsA indicated that plant oils worked in another way rather than being the substrates.

Taken the above results, soybean oil played a significant role in improving biomass and specific production rate. However, soybean oil might not play its role in the lipid metabolic pathway and more intercellular metabolic details still remained to be investigated. To this end, a combined proteomic and metabolic analysis was implemented to further reveal the role of soybean oil in [(4′-OH)MeLeu]^4^-CsA overproduction.

### Comparative proteomic and metabolomic analyses in response to the soybean oil addition

#### Comparative proteomic profile analysis

**Fig. 3:**
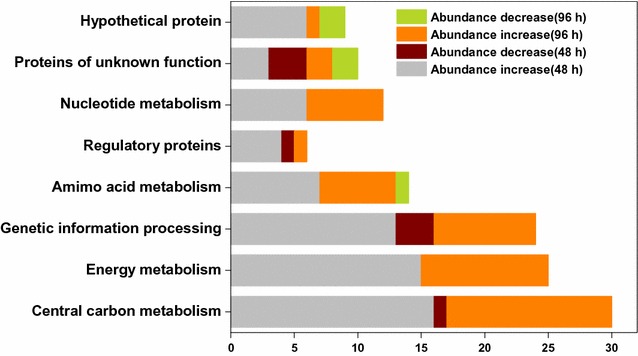
Protein distribution with significant changes in each functional category at 48 and 96 h respectively. 2-DE profiles of *N. dietziae* were presented in Additional file 1: Figure S4 and the significantly differential proteins and their characteristics were listed in Table 1

Protein extracts from both conditions were sampled at 48 h and 96 h and then subjected to 2-DE. The results of gel electrophoresis were presented in Additional file 1: Figure S4 and 2-DE could effectively separate most of the proteins, indicating the feasibility of our experimental methods. In this study, a total of 95 protein spots in the 2-DE gels with significantly differential expressions (fold change >1.5) were identified (Table 1). They were classified into eight functional groups by their cellular roles, mainly including central carbon metabolism, energy metabolism, genetic information processing, amino acid metabolism, regulatory proteins, nucleotide metabolism, proteins of unknown function and hypothetical proteins. Figure 3 presented the protein distribution at the sampling time with different abundances, and most of the proteins in each group were present at higher levels under the MO condition, suggesting a comprehensive strengthening effect of soybean oil on the whole cell metabolism.

**Table 1:** Differentially expressed proteins identified by MALDI-TOF/TOF–MS under the soybean oil and the control conditions

Spot no.^a^	Protein name	Species	NCBI accession no.^b^	Protein MW^c^	Protein *PI*	Protein score^d^	Protein score C.I. %	MO/MC (48 h)^e^	MO/MC (96 h)
1	Glucose-6-phosphate dehydrogenase	*Nonomuraea* sp. *SBT364*	gi|898235202	56.92	5.96	138	100	1.67	0.56
2	Phosphogluconate dehydratase	*Nonomuraea* sp. *SBT364*	gi|898253514	68.06	5.8	67	99.5	Loss	1.64
3	Enolase	*Streptosporangium amethystogenes*	gi|664384366	45.33	4.53	72	99.76	1.78	1.89
4	3-phosphoglycerate dehydrogenase	*Nonomuraea candida*	gi|759933157	55.53	4.99	135	100	1.96	1.87
5	Dihydrolipoamide dehydrogenase	*Nonomuraea candida*	gi|759953882	47.83	5.47	62	98.23	1.93	1.21
6	6-phosphofructokinase	*Nonomuraea coxensis*	gi|648522261	36.66	5.53	125	100	2.17	1.74
7	Pyruvate dehydrogenase	*Nonomuraea candida*	gi|759929120	102.11	5.83	146	100	2.35	1.85
8	Pyruvate kinase	*Nonomuraea candida*	gi|759954395	51.48	5.94	118	100	2.05	1.78
9	Malate dehydrogenase	*Nonomuraea* sp. *SBT364*	gi|898218461	34.1	4.85	65	99.23	1.75	1.64
10	2-oxoglutarate ferredoxin oxidoreductase subunit alpha	*Nonomuraea* sp. *SBT364*	gi|898257150	65.95	5.34	76	99.94	1.68	0.77
11	Citrate synthase	*Nonomuraea candida*	gi|759936001	40.74	5.45	67	99.25	3.32	1.71
12	Succinyl-CoA synthetase subunit alpha	*Nonomuraea* sp. *SBT364*	gi|898242487	30.38	6.1	131	100	2.45	1.78
13	Alpha-ketoglutarate decarboxylase	*Nonomuraea candida*	gi|759939264	134.27	5.91	103	100	2.19	1.62
14	Cyclopropane-fatty-acyl-phospholipid synthase	*Nonomuraea candida*	gi|759945365	46.94	6.07	67	99.35	1.62	0.72
15	Cytochrome P450 hydroxylase sb15	*Nonomuraea dietziae*	gi|445067401	51.04	8.82	34	99.11	1.73	1.67
16	2-isopropylmalate synthase	*Nonomuraea* sp. *SBT364*	gi|898222194	63.16	5.04	187	100	1.25	1.84
17	Cytochrome P450 hydroxylase sb8	*Nonomuraea dietziae*	gi|445067389	44.43	5.23	25	97.84	1.85	0.82
18	Cytochrome P450 hydroxylase sb17	*Nonomuraea dietziae*	gi|445067405	43.62	5.19	25	97.37	2.06	0.71
19	Cytochrome P450 hydroxylase sb2	*Nonomuraea dietziae*	gi|445067377	25.04	5.5	33	98.85	1.76	1.22
20	Cytochrome P450 hydroxylase sb20	*Nonomuraea dietziae*	gi|445067407	42.77	5.05	34	99.15	1.95	1.65
21	NADH dehydrogenase	*Nonomuraea candida*	gi|759944851	49.06	5.22	112	100	3.39	0.97
22	Acyl-CoA thioesterase	*Alicyclobacillus acidoterrestris*	gi|916582360	17.26	5.58	89	96.85	0.61	1.68
23	NADP oxidoreductase	*Nonomuraea coxensis*	gi|916408869	47.19	6.08	71	99.79	1.87	2.15
24	Flavoprotein disulfide reductase	*Streptosporangium amethystogenes*	gi|664385831	48.56	5.37	69	99.55	2.08	1.75
25	NADH dehydrogenase subunit F	*Streptosporangium roseum*	gi|502651094	46.67	5.5	115	100	0.68	1.94
26	FAD-linked oxidase	*Nonomuraea* sp. *SBT364*	gi|898280523	55.07	5.87	92	100	1.72	1.98
27	Flavoprotein oxidoreductase	*Streptosporangium roseum*	gi|502655712	48.81	5.58	60	99.55	1.79	1.73
28	Transcription termination factor NusA	*Nonomuraea coxensis*	gi|522034561	36.32	5.43	87	99.99	2.27	1.17
29	MFS transporter	*Streptosporangium amethystogenes*	gi|664381342	79.76	6.34	57	97.78	1.64	1.32
30	Thiosulfate sulfurtransferase	*Nonomuraea coxensis*	gi|522033492	31.21	4.81	62	98.19	1.74	1.94
31	Sulfonate ABC transporter ATP-binding protein	*Nonomuraea candida*	gi|759950693	25.17	7	55	97.5	1.80	1.68
32	ATP-binding protein	*Nonomuraea coxensis*	gi|648523005	48.21	5.74	150	100	1.67	0.88
33	S-adenosylmethionine synthetase	*Streptosporangium amethystogenes*	gi|664383325	42.57	4.98	120	100	2.04	1.17
34	S-adenosyl-l-homocysteine hydrolase	*Nonomuraea coxensis*	gi|522030148	52.1	5.33	91	100	1.66	4.28
35	Glutamate synthase	*Nonomuraea coxensis*	gi|703367593	160.46	5.48	79	99.96	2.47	0.62
36	Phenylalanine–tRNA ligase subunit alpha	*Nonomuraea coxensis*	gi|522035904	38.25	5.4	93	100	1.74	1.32
37	Aspartyl/glutamyl-tRNA amidotransferase subunit B	*Streptosporangium roseum*	gi|502658302	54.44	5.13	117	100	1.88	2.38
38	Proline–tRNA ligase	*Nonomuraea candida*	gi|759956543	63.49	5.24	107	100	11.29	2.46
39	Tryptophanyl-tRNA synthetase	*Nonomuraea* sp. *SBT364*	gi|898218468	37.51	6.21	100	100	0.45	1.46
40	Cysteine synthase	*Nonomuraea coxensis*	gi|522035423	34.18	5.47	70	99.75	2.45	1.45
41	ATPase AAA	*Lachnoanaerobaculum* sp. *ICM7*	gi|497349325	83.91	5.1	186	100	1.82	1.63
42	Urocanate hydratase	*Nonomuraea coxensis*	gi|703370540	59.91	5.54	75	99.9	1.71	2.08
43	Histidine ammonia-lyase	*Nonomuraea* sp. *SBT364*	gi|898282330	53.36	5.34	67	99.4	1.74	0.97
44	Inosine-5-monophosphate dehydrogenase	*Nonomuraea* sp. *SBT364*	gi|759938464	39.48	5.74	150	100	2.02	1.76
45	Polynucleotide phosphorylase	*Nonomuraea coxensis*	gi|522034552	84.03	5.11	158	100	0.76	1.62
46	Elongation factor Ts	*Streptosporangium roseum*	gi|502652347	30.18	5.27	121	100	9.17	0.89
47	DNA-directed RNA polymerase subunit alpha	*Alkalibacillus haloalkaliphilus*	gi|515752615	35.17	4.8	282	96.64	1.61	0.69
48	DNA polymerase III subunit beta	*Nonomuraea* sp. *SBT364*	gi|898216335	40.14	4.79	215	100	1.67	0.73
49	30S ribosomal protein S2	*Nonomuraea* sp. *SBT364*	gi|898235817	34.47	5.02	161	100	1.78	1.62
50	Transcription termination factor Rho	*Nonomuraea* sp. *SBT364*	gi|898215154	73.6	8.73	140	100	1.62	0.49
51	Dihydrolipoyl dehydrogenase	*Nonomuraea* sp. *SBT364*	gi|898266057	49.7	5.51	126	100	1.74	1.85
52	Peptide chain release factor 2	*Nonomuraea candida*	gi|759935368	41.51	4.69	109	100	0.65	1.94
53	Molecular chaperone GroEL	*Nonomuraea candida*	gi|759948935	57.13	4.84	166	100	0.42	1.32
54	Histidine kinase	*Nonomuraea coxensis*	gi|703366436	148.43	4.97	267	100	1.74	1.36
55	Elongation factor P	*Nonomuraea coxensis*	gi|522034280	19.93	5.00	130	100	0.45	0.90
56	ATP-dependent Clp protease proteolytic subunit	*Nonomuraea candida*	gi|759955675	23.2	5.03	108	100	0.70	1.67
57	Two-component system sensor histidine kinase	*Nonomuraea candida*	gi|759950142	37.01	9.94	59	96.39	1.68	1.51
58	Proteasome subunit alpha	*Nonomuraea coxensis*	gi|916409057	30.52	4.99	91	100	1.71	0.76
59	ATPase AAA	*Nonomuraea* sp. *SBT364*	gi|898282349	65.47	5.04	186	100	1.07	1.75
60	Carbamoyl phosphate synthase large subunit	*Nonomuraea* sp. *SBT364*	gi|898274197	117.27	4.79	139	100	1.64	0.96
61	LysR family transcriptional regulator	*Streptosporangium roseum*	gi|759969058	33.7	6.28	62	98.03	0.93	1.05
62	DNA-binding response regulator	*Nonomuraea coxensis*	gi|522033451	25.6	5.17	69	99.64	1.69	0.90
63	Hypothetical protein	*Kiloniella laminariae*	gi|759750848	27.62	10.15	115	100	2.69	0.81
64	Gamma-aminobutyraldehyde dehydrogenase	*Nonomuraea candida*	gi|759930674	49.16	5.26	107	100	1.68	1.74
65	Monooxygenase	*Nonomuraea candida*	gi|759940588	49.69	6.00	62	98.31	2.14	1.76
66	GntR family transcriptional regulator	*Bacillus cereus*	gi|872654957	54.7	9.01	86	96.79	0.90	1.62
67	Molecular chaperone	*Nonomuraea candida*	gi|759944965	67.07	4.81	318	100	0.42	1.32
68	Cyclophilin	*Nonomuraea candida*	gi|759934622	19.5	5.96	55	96.73	1.61	0.74
69	Bifunctional 5,10-methylene-tetrahydrofolate dehydrogenase	*Nonomuraea coxensis*	gi|648522670	29.34	5.4	86	99.99	1.65	1.35
70	Alanine dehydrogenase	*Nonomuraea candida*	gi|759942142	38.78	5.78	80	99.97	1.94	0.47
71	Hydrolase	*Nonomuraea candida*	gi|759945898	46.66	4.64	77	99.93	0.49	1.64
72	RNase J family beta-CASP ribonuclease	*Streptosporangium amethystogenes*	gi|664377501	60.89	5.68	138	99	1.25	1.68
73	Methylmalonyl-CoA mutase	*Streptosporangium roseum*	gi|502654007	116.92	5.5	67	99.3	1.32	1.86
74	Phage-shock protein	*Nonomuraea candida*	gi|759953906	29.75	5.4	107	99.78	0.45	0.97
75	Citrate synthase 2	*Nonomuraea* sp. *SBT364*	gi|898229311	39.82	5.97	159	100	1.71	1.05
76	Adenylosuccinate synthetase	*Streptosporangium amethystogenes*	gi|664389689	46.43	5.85	134	100	1.84	0.85
77	NDP-hexose 4-ketoreductase	*Streptosporangium roseum*	gi|665581403	92.72	5.73	252	100	1.63	0.31
78	Vitamin B12-dependent ribonucleotide reductase	*Nonomuraea candida*	gi|759929294	103.74	5.64	99	100	2.20	1.69
79	DNA-binding response regulator	*Nonomuraea* sp. *SBT364*	gi|898242523	22.86	5.87	157	100	3.99	0.89
80	Acetyltransferase	*Nonomuraea candida*	gi|759934501	20.5	8.7	56	98.61	0.43	1.85
81	Peptidase M48	*Nonomuraea* sp. *SBT364*	gi|898280126	38.21	5.89	204	99.91	0.43	1.31
82	Argininosuccinate lyase	*Streptosporangium roseum*	gi|759973125	53.3	5.43	59	96.67	2.44	1.32
83	GlmZ(sRNA)-inactivating NTPase	*Streptosporangium roseum*	gi|665597475	31.53	5.55	110	100	0.16	0.73
84	Adenylosuccinate lyase	*Nonomuraea candida*	gi|759952039	51.67	6.18	141	100	0.78	1.69
85	Sulfate adenylyltransferase	*Nonomuraea coxensis*	gi|522034000	46.94	5.6	194	100	1.64	0.83
86	Serine hydroxymethyltransferase	*Nonomuraea coxensis*	gi|522029537	44.16	6.01	162	100	1.74	1.64
87	Glycosyl transferase	*Streptosporangium amethystogenes*	gi|664380822	33.06	9.09	56	96.21	1.07	1.82
88	Hypothetical protein	*Nonomuraea coxensis*	gi|918028067	34.38	5.27	61	97.09	1.25	0.48
89	Hypothetical protein	*Ruminococcaceae bacterium AE2021*	gi|522080859	26.13	9.69	103	100	2.06	0.73
90	Hypothetical protein	*Borrelia afzelii*	gi|522034300	51.1	4.66	61	97.35	0.74	1.75
91	Hypothetical protein	*Streptosporangium roseum*	gi|648523244	38.07	5.88	119	100	1.51	0.42
92	Hypothetical protein	*Streptosporangium roseum*	gi|502657796	77.87	6.54	66	99.1	1.94	1.04
93	Hypothetical protein	*Streptomyces fradiae*	gi|921240484	26.09	5.42	103	100	1.65	0.77
94	Hypothetical protein	*Nonomuraea* sp. *SBT364*	gi|759929790	14.37	4.86	62	98.15	1.88	0.64
95	Hypothetical protein	*Sunxiuqinia dokdonensis*	gi|501669599	85.44	8.09	97	98	1.82	0.78

^a^Spot number of the differentially protein in Fig. 2

^b^Accession numbers in the NCBInr database

^c^Theoretical molecular weight (kDa) and isoelectric point (pI)

^d^Mascot score from the NCBInr

^e^Mean fold change is the ratio of protein abundance between medium MO and MC at 48 and 96 h, respectively

#### Comparative metabolic profile analysis

**Fig. 4:**
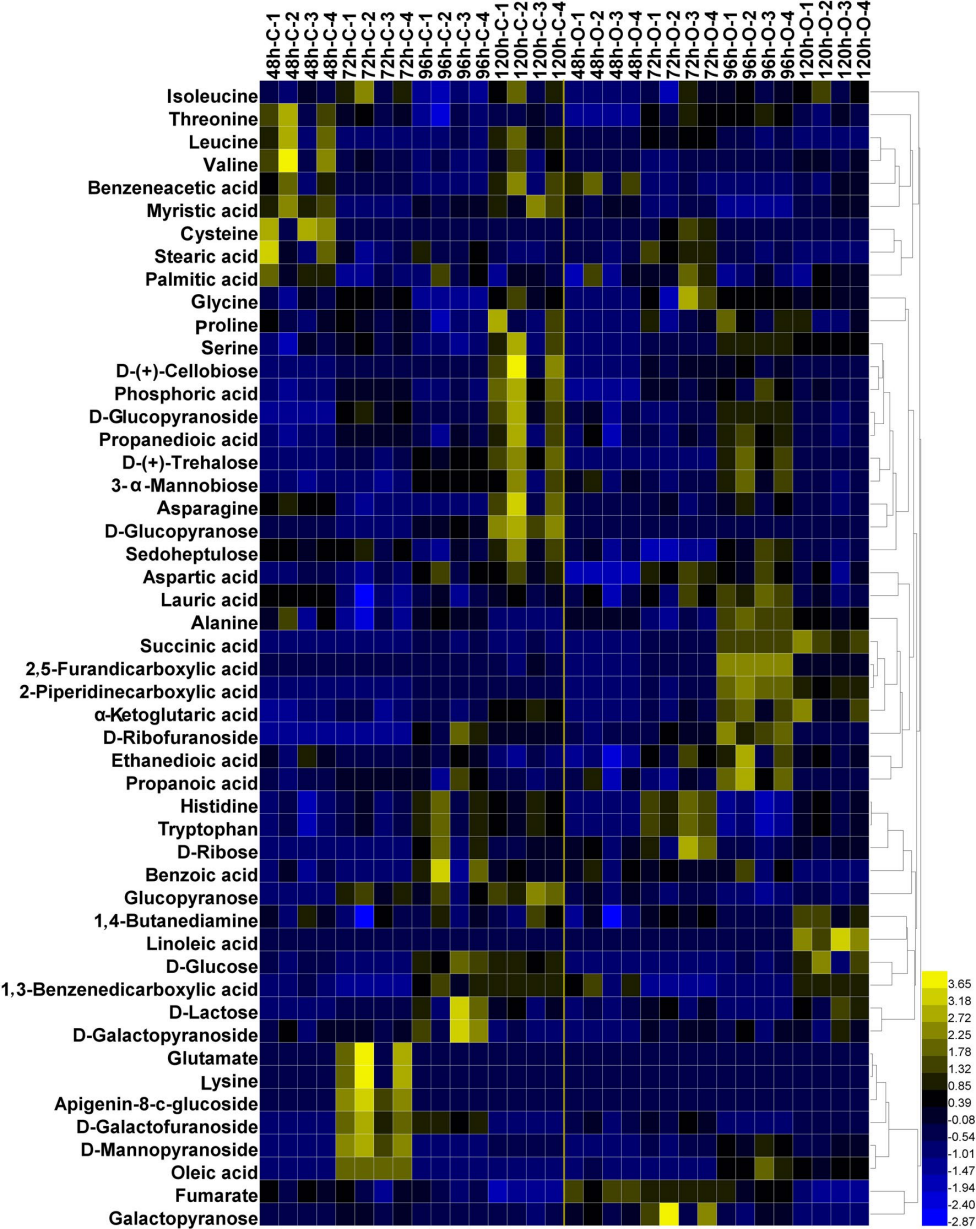
Heatmap of intracellular metabolite abundances at each sample time. Relative abundances of intracellular metabolites are processed by zero-mean normalization. All the metabolites are ordered by hierarchical cluster analysis

Intercellular metabolites of *N. dietziae* (48, 72, 96 and 120 h) in the medium MO and MC with different [(4′-OH)MeLeu]^4^-CsA producing capabilities were analyzed by GC–MS. As a result, a total of 50 intracellular metabolites were identified, including amino acids, organic acids, sugars and fatty acids. Abundance changes of metabolites are depicted by the heat map in Fig. 4.

The metabolomic profiling showed obvious differences in tricarboxylic acid (TCA) cycle and amino acid metabolism. More specifically, succinic acid increased to 2.8- and 5.5-fold of that in the medium MC at 48 and 96 h, respectively. Similar change patterns also exhibited in fumarate (1.4- and 1.2-fold vs. the control) and α-ketoglutaric acid (1.2- and 2.2-fold vs. the control). Although the abundances of these metabolites decreased gradually along the fermentation process, they were still much higher than the control, indicating the enhancement of TCA cycle in medium MO. On the contrary, the abundance of amino acids such as threonine, glutamate, lysine, valine and leucine were relatively lower in medium MO. Particularly, both glutamate and lysine decreased by 78% at 72 h. A reasonable explanation was that large amount of amino acids was effectively consumed to synthesize cellular building blocks for cell growth.

**Fig. 5:**
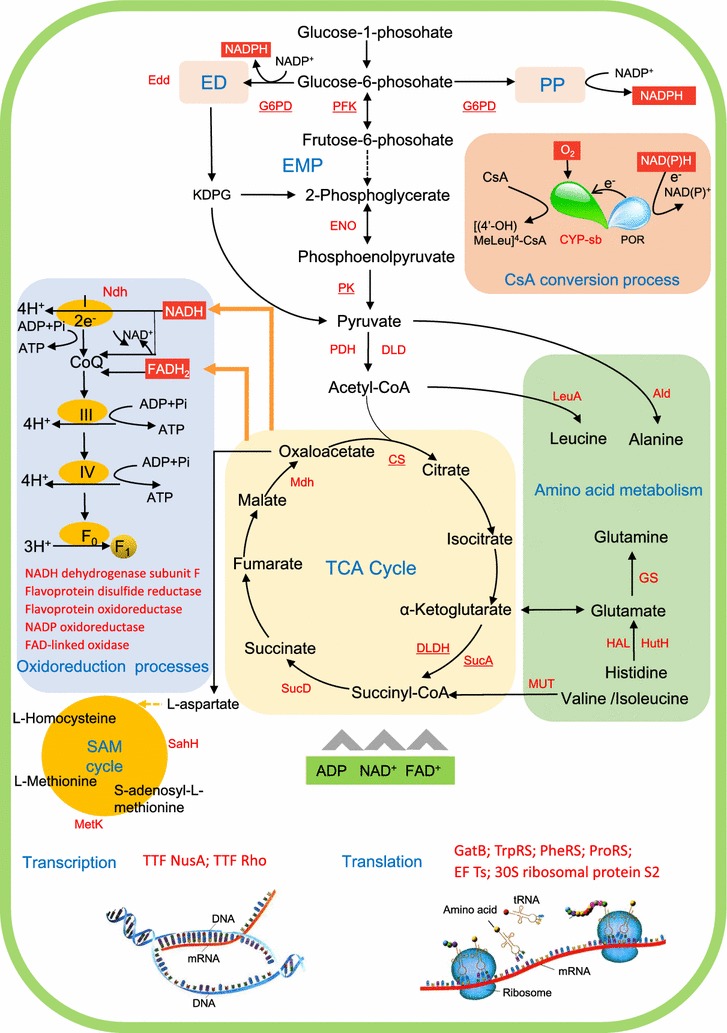
Scheme of metabolic pathways in [(4′-OH)MeLeu]^4^-CsA production under MO condition. Enzymes in *red font* are activated under MO condition and the rate-limiting enzymes are *underlined* besides. Regions of *different colors* represent different metabolic modules. *G6PD* glucose-6-phosphate dehydrogenase, *Edd* phosphogluconate dehydratase, *KDPG* 2-keto-3-deoxy-6-phosphogluconate, *PFK* 6-phosphofructokinase, *ENO* enolase, *PK* pyruvate kinase, *DLD* dihydrolipoamide dehydrogenase, *PDH* pyruvate dehydrogenase, *CS* citrate synthase, *DLDH* dihydrolipoyl dehydrogenase, *SucA* alpha-ketoglutarate decarboxylase, *SucD* succinyl-CoA synthetase alpha subunit, *Ndh* NADH dehydrogenase, *Ald* alanine dehydrogenase, *LeuA* 2-isopropylmalate synthase, GS glutamate synthase, *HutU* urocanate hydratase, *HAL* histidine ammonia-lyase, *MUT* methylmalonyl-CoA mutase, *MetK* S-adenosylmethionine synthetase, *SahH* S-adenosyl-l-homocysteine hydrolase, *CYP* cytochrome P450 hydroxylase, *POR* P450 oxidoreductase, *PheRS* phenylalanine–tRNA ligase, *GatB* aspartyl/glutamyl-tRNA amidotransferase subunit B, *ProRS* proline–tRNA ligase, *TrpRS* tryptophanyl-tRNA synthetase, *EF-Ts* elongation factor Ts

Based on the proteomic and metabolomic results, we therefore summarized the metabolic profile in Fig. 5, which visually described the intracellular metabolic pathway in response to the exogenous soybean oil. In this model, most proteins with high levels (in red font) were mainly involved in the central carbon metabolism, amino acid metabolism, genetic information processing and oxidation–reduction process. As regarding to the hydroxylation of CsA to [(4′-OH)MeLeu]^4^-CsA, it was directly mediated by CYPs and P450 oxidoreductase (POR) along with the formation of water and NAD(P)^+^, which was seemingly uncorrelated to other intracellular metabolic activities. Subsequently, pathway analysis was implemented to dissect the effects of soybean oil on the metabolism and regulation of *N. dietziae* in detail.

#### Central carbon metabolism and amino acid metabolism

Central carbon metabolism represents the backbone of the cellular metabolism and provides the precursors required for the cell growth and the synthesis of target products. As shown in Fig. 5, it was worth noting that some key enzymes involved in glycolytic pathway, such as 6-phosphofructokinase (Spot 6, PFK) and pyruvate kinase (Spot 8, PYK) were both present at higher levels. Different from many other actinomycetes, PFK of *Nonomuraea* is not allosterically regulated by ATP, AMP, ADP, or phosphoenolpyruvate and pyruvate, but controlled by the availability of pyrophosphate (PPi) produced in nucleic acid and protein biosynthesis and in the cycling between glycogen and glucose-1-phosphate [23]. Additionally, the activity of PPi-dependent PFK is reversible, suggesting that *N. dietziae* had a flexible glycolytic pathway as the regulatory node. Apart from PFK, dihydrolipoamide dehydrogenase (Spot 5, DLD), pyruvate dehydrogenase (Spot 7, PDH) and enolase (Spot 3, ENO) also showed higher levels under the MO condition, suggesting a strengthened Embden–Meyerhof–Parnas (EMP) pathway.

In regard to TCA cycle, citrate synthase (Spot 11, CS), which converts acetyl-CoA and oxaloacetate to citrate in the initial step and controls flux into the TCA cycle [24], showed a higher level (3.23-folds) under the MO condition while the increase in the level of malate dehydrogenase (Spot 9, Mdh) could supply more oxaloacetate that reacts with acetyl-CoA [25]. In addition, the higher levels of alpha-ketoglutarate decarboxylase (Spot 13, SucA) and dihydrolipoyl dehydrogenase (Spot 51, DLDH) further improved the metabolic rate of TCA cycle [26]. As a consequence, TCA-related metabolites, such as fumarate, α-ketoglutaric acid and succinic acid, were all present at higher levels (Fig. 4), indicating that the TCA cycle was active under the MO condition from both proteomic and metabolomic insights.

Additionally, glucose-6-phosphate dehydrogenase (Spot 1, G6PD), the first key enzyme of pentose phosphate (PP) pathway, had a higher level (1.67-fold) at 48 h under the MO oil condition, but at 96 h the abundance of G6PD decreased to 56% of the control. G6PD-catalyzed reaction dictates and limits the fluxes between Embden–Meyerhof–Parnas (EMP) and PP pathways [27], and the PP pathway oxidizes glucose to generate NADPH for reductive biosynthesis reactions and ribose-5-phosphate for the synthesis of the nucleotides [28]. The decreased level of G6PD under the MO condition restricted the specific cell growth (Fig. 1, 96 h) and the specific production rate of [(4′-OH)MeLeu]^4^-CsA (Fig. 1, 96 h) since the cofactor NADPH also participated in the CsA hydroxylation process in addition to biomass accumulation.

Another significantly changed protein, phosphogluconate dehydratase (Spot 2, Edd), was only detected at 96 h and had a 64% increase after the soybean oil addition. Gunnarsson et al. had identified the ED pathway in *Nonomuraea* sp. by the ^13^C labelling-based method [29]. ED pathway catabolizes glucose to pyruvate by using the 6-phosphogluconate dehydratase (Edd) and 2-keto-3-deoxyphosphogluconate aldolase (KDPG aldolase), which connects the EMP, PP pathways and TCA cycle, and tunes the carbon flux distribution, the production of ATP, reducing equivalent for cell growth. As the first critical enzyme of ED pathway, the higher level of Edd under the MO condition probably indicated a more robust sugar metabolism at the end of fermentation than the control.

Amino acid metabolism is essential for cell growth by supplying the building blocks and metabolic intermediates along with EMP, TCA and PPP. In this study, three proteins involved in glutamate synthesis, i.e. Spot 42 (urocanate hydratase, HutU), Spot 43 (histidine ammonia-lyase, HAL) and Spot 35 (glutamate synthase, GS), as well as some other amino acid metabolism-related proteins (Spot 16, 2-isopropylmalate synthase, LeuA, Spot 40, cysteine synthase, CysO) had significantly higher levels in the medium MO. Although abundances of glutamate, leucine and alanine were much lower in the medium MO than that in the medium MC (Fig. 4), it could probably be resulted from the effective utilization for protein synthesis and cell growth. In addition, two proteins in S-adenosyl-l-methionine (SAM) cycle, i.e. Spot 33 (SAM synthetase, MetK) and Spot 34 (S-adenosyl-l-homocysteine hydrolase, SahH) were detected at higher abundance in the medium MO, which were involved in the synthesis of Fe–S protein clusters and methionine [30].

Therefore, it can be concluded that addition of soybean oil could significantly upregulate the intracellular central carbon metabolism and amino acid metabolism, which supplies a more suitable intracellular environment for maintaining the cell robustness and promoting the [(4′-OH)MeLeu]^4^-CsA overproduction.

#### Genetic information processing and nucleotide metabolism

Bacterial growth is directly correlated to the synthesis of protein and DNA. In this study, the higher abundances of phenylalanine–tRNA ligase (PheRS, Spot 36), aspartyl/glutamyl-tRNA amidotransferase subunit B (GatB, Spot 37), proline–tRNA ligase (ProRS, Spot 38) and tryptophanyl-tRNA synthetase (TrpRS, Spot 39) were observed under the MO condition, indicating that aminoacyl-tRNA biosynthesis was activated. Additionally, elongation factor Ts (EF-Ts, Spot 46) and 30S ribosomal protein S2 (Spot 49) were also present at higher levels compared with the control. EF-Ts mediates the regeneration of EF-Tu-GDP complex, which catalyzes the addition of aminoacyl-tRNA into ribosome [31, 32]. Meanwhile, DNA-directed RNA polymerase subunit alpha (Spot 47), DNA polymerase III subunit beta (Spot 48), transcription termination factor NusA (Spot 28) and transcription termination factor Rho (Spot 50), were present at higher levels. They are involved in the transcription and replication processes and inosine-5-monophosphate dehydrogenase (Spot 44) provides precursors (guanine) for RNA and DNA synthesis [33]. Higher levels of the above proteins could efficiently enhance the cell growth, leading to a higher specific growth rate under the MO condition as shown in Fig. 1.

#### Cytochrome P450 (CYP) and energy metabolism

CYPs belong to a family of terminal monooxygenases that transfer one oxygen atom to X–H bonds (X:-C,-N, S) of a substrate with the concomitant reduction of the other oxygen atom to water [14]. CYPs directly participate in the bioconversion of CsA to [(4′-OH)MeLeu]^4^-CsA. In this study, five CYP isoforms (Spot 15, 17–20) were identified and most of them showed higher abundance compared with the control (Table 1). Proteins involved in energy metabolism, especially the redox reaction, displayed a significant improvement (Table 1) under the MO condition. 15 of 16 proteins involved in energy metabolism had higher levels at 48 h, and 10 proteins demonstrated the similar expression pattern at 96 h (Table 1). In addition, proteins related to the electron transfer chain showed significantly higher levels in the medium MO. In particular, NADH dehydrogenase (Spot 21), the first proton pump in oxidative phosphorylation, showed a 2.4-fold higher abundance than the control at 48 h. Throughout the fermentation process, the abundances of NADH dehydrogenase (Spot 21), FAD-linked oxidase (Spot 26) and flavoprotein oxidoreductase (Spot 27) were significantly increased (>3.1-folds) in the medium MO. It is worth noting that NADH dehydrogenase showed the maximum change (20.9-folds) among all the identified proteins. Since the growth of *Nonomuraea* sp. is strictly dependent on the aerobic metabolism and oxidative phosphorylation [34], as the key members of electron transfer chain, these proteins with higher levels would enhance the accessibility and the turnover rate of NADH and FADH to CYPs so as to improve the hydroxylation activity [15].

### Transcription profiles of the CYPs under the MO condition and the control condition

Since CYPs directly participates in the monooxygenation reaction, the transcriptional level of CYPs is one of the key factors limiting the conversion efficiency. In this study, all the transcriptional expression of CYPs were detected to investigate the impact of soybean oil addition on the CYPs. Previous work has reported that there are 21 species of CYP-sb, among which sb21 plays a leading role in the bioconversion of CsA, and then is CYP-sb22, CYP-sb13, CYP-sb7, and CYP-sb8, etc. according to the single-gene knockout results [14].

The sampling time points 36 h (12 h after soybean oil addition) and 48 h (24 h after soybean oil addition) were selected since the greatest specific production rate appeared before 48 h under both conditions. As shown in Table 2, only 11 CYP genes were detected under both conditions while the other 10 genes were detected only in certain conditions. At 36 h, not only the number of the genes but also the transcription abundance of the CYPs in the soybean oil group were significantly increased compared with the control group (Table 2), which could explain why the specific production rate of the soybean oil group was much higher than that of the control group at 36 h.

**Table 2:** Transcription profiles of CYPs under the soybean oil and the control conditions

Gene name	∆Ct_C1_				−∆∆Ct	
	C1	C2	S1	S2	S1 vs. C1	S2 vs. C2
CYP-sb1	19.08	16.06	ND	18.64	–	−2.58
CYP-sb10	ND	17.28	ND	ND	–	–
CYP-sb11	13.01	12.45	13.00	13.36	0.01	−0.91
CYP-sb12	6.00	9.23	3.32	8.82	2.68	0.41
CYP-sb13	20.63	20.80	18.18	11.33	2.45	9.47
CYP-sb15	7.05	5.38	6.39	8.24	0.66	−2.86
CYP-sb16	ND	20.66	ND	ND	–	–
CYP-sb17	ND	17.08	14.53	10.85	–	6.23
CYP-sb2	9.16	10.90	9.79	12.55	−0.63	−1.65
CYP-sb20	9.13	10.70	9.54	8.26	−0.41	2.44
CYP-sb21	8.22	11.11	4.28	4.96	3.94	6.15
CYP-sb22	9.78	10.04	7.20	10.83	2.58	−0.79
CYP-sb23	ND	12.28	13.87	12.59	–	−0.31
CYP-sb24	ND	ND	11.94	12.56	–	–
CYP-sb3-1	ND	15.17	ND	ND	–	–
CYP-sb3-2	16.76	11.63	12.07	8.40	4.69	3.23
CYP-sb4	ND	22.32	ND	11.90	–	10.42
CYP-sb6	19.99	ND	14.38	17.59	5.61	–
CYP-sb7	ND	12.85	17.41	ND	–	–
CYP-sb8	12.15	11.64	8.32	12.31	3.83	−0.67
CYP-sb9	9.35	8.91	7.89	10.13	1.46	−1.22

C1, C2, S1 and S2 stand for the samples of 36 and 48 h under the control condition and the soybean oil condition, respectively

∆Ct: The difference value of Cycle Time between the targeted gene and the reference gene. ∆Ct = *Ct(Targeted gene)* − *Ct(16S rDNA)*. A lower ∆Ct represents a higher relative transcription level. −∆∆Ct = log_2_(fold change)

Each data was calculated the mean value of six samples (three biological repeations and two technical repeations)

*ND* not detected

At 48 h, the number of the detected CYPs in the control group was larger than that of the soybean oil group, but the transcription levels of the crucial CYP gene sb21, sb13 and sb3-2 were still much lower than the soybean oil group, which could explain the phenomenon that in Fig. 1c the specific production rate of the control group at 48 h was increased, but was still lower than that in the MO condition. In addition, the upregulation of the transcription level of the CYPs under the MO condition also implied the formation of the CYP-CsA complex since the TMC (total mole concentration) in the fermentation broth reached bottom at 48 h and the TMC in the MO medium was lower than that in the MC medium as shown in Fig. 1d.

Interestingly, the transcription level of CYP-sb21 in the control group greatly reduced at 48 h (Table 2) but the catalytic efficiency reached the maximum (Fig. 1c). Although sb21 played a leading role in the conversion of CsA to [(4′-OH)MeLeu]^4^-CsA [14], yet the role of other CYPs should not be neglected because the expression of sb3-1, sb4, sb7, sb10, sb16, sb17, sb23 and sb24, and the upregulation of sb3-2, sb8, sb9, sb11 and sb15 counteracted the effect of the down-regulated sb21.

### Proposed metabolic mechanism of [(4′-OH)MeLeu]^4^-CsA overproduction under the MO condition by *N. dietziae*

Soybean oil could improve the production of antibiotics, such as tetracycline, cephamycin C and tacrolimus [18–20], but the specific mechanism was not always the same. Soybean oil was found to enhance tetracycline production by extracting antibiotic into the oil phase of the culture broth, thereby relieving product inhibition and decreasing damage to microbial cells by foam formation [18]. While in cephamycin C production, soybean oil mainly functioned as a carbon source for *Streptomyces* sp. p6621 fermentation [19]. Additionally, soybean oil could induce the expression of lipase to produce CoA-esters, the precursors of FK506 by *S. tsukubaensis* [20, 22]. These three products have a similarity that they are produced by the de novo synthesis pathway. However, [(4′-OH)MeLeu]^4^-CsA is converted from CsA by the monooxygenation reaction and is directly associated with CYPs, POR (cytochrome P450 reductase), oxygen and reducing equivalent. Thus, the role of soybean oil herein is probably not one of the above mentioned. In this study, the apparent catalytic efficiency (or conversion rate) is closely associated with two factors, i.e., the conversion capacity per unit of the biomass (μ_p_) and the quantity of biomass (DCW). μ_p_ is the intrinsic property which is dependent on the abundance of CYPs, PORs and the accessibility of reducing equivalent supplied by the cell metabolism. The synthesis of biomass relies on the abundance of intracellular building blocks, the rate of genetic information processing and the energy availability (ATP and reducing equivalent).

On one hand, the proteomic analysis showed that the significantly changed proteins were involved in EMP, TCA cycle, amino acid metabolism and redox process, thereby enhancing the flux into central carbon metabolism and supplying sufficient cellular building blocks, ATP and reducing equivalents for maintaining the cell robustness and promoting the hydroxylation of CsA. Meanwhile, the improvement of transcription and translation process helps to accelerate the protein synthesis and mycelium growth. Moreover, metabolomic analysis indicated that soybean oil had a great effect on amino acid metabolism and tricarboxylic acid cycle.

On the other hand, the transcriptional analysis of all the CYPs under both conditions confirm that soybean oil can strengthen the CYP system for the conversion of CsA to [(4′-OH)MeLeu]^4^-CsA. Additionally, the enhanced hydroxylation ability is not only dependent on the elevated expression of CYP-sb21, but also on other CYPs, such as CYP-sb13 and CYP-sb8, although they work with different degrees. These findings demonstrate that the CsA conversion process is under a sophisticated and systematic regulation, although it’s a simple in vivo monooxygenation reaction.

Another phenomenon is that besides the soybean oil, some other plant oils, such as corn oil and peanut oil, could also exert a positive influence on the [(4′-OH)MeLeu]^4^-CsA improvement, implying a common feature of these plant oils. In the proteomic analysis, it was noteworthy that no differentially expressed proteins were observed in lipid metabolism (Table 1), implying that the major contribution of soybean oil may not be related to lipid metabolism. As regarding to the metabolomic data, oleic acid, stearic acid, and myristic acid were more abundant in MC samples, especially in the initial fermentation stage (48 and 72 h) (Fig. 4). These results demonstrated that lipid metabolism had not been activated under the MO condition. Moreover, the main hydrolysates of soybean oil did not strengthen the CsA conversion ability (Fig. 2). All these results indicated that plant oils could influence the fermentation characteristics. A reasonable explanation was that plant oils increased the oxygen transfer efficiency due to their lower polarity and the stronger oxygen-carrying capacity compared with water [35], thus strengthening the oxygen supply for intracellular redox metabolism and CsA conversion process, just as shown in metabolic pathway analysis in response to soybean oil.

## Conclusions

In summary, a systematic comparative proteomic and metabolomic analysis was successfully conducted to gain insights into the role of soybean oil in improving [(4′-OH)MeLeu]^4^-CsA production by *N. dietziae*. Soybean oil could strengthen the primary pathways and the CYP system of *N. dietziae*, thereby improving the rate of biomass synthesis and the hydroxylation efficiency. The omics-based analysis provides a number of intracellular biomarkers, and is a starting point for exploring the regulatory mechanism between cell growth and [(4′-OH)MeLeu]^4^-CsA production, which will be a guidance for the further metabolic engineering of this strain.

## Methods

### Microorganism and cultivations

*Nonomuraea dietziae* used in this study was stocked in our laboratory and cultivated on ISP-2 agar slant [36]. Seed medium was prepared as Zhang’s work [37]. Production medium: 20 g/L glucose, 3 g/L yeast extract, 10 g/L peptone, 12 g/L dextrin, 15 g/L corn steep liquor and pH 6.5. *N. dietziae* spores were washed from the fresh agar slant by 5 mL sterile 0.9% NaCl solution and transferred into 100 mL seed medium of a 500 mL Erlenmeyer flask and then incubated at 28 °C, 220 rpm for 72 h. The [(4′-OH)MeLeu]^4^-CsA production was carried out in 50 mL medium of a 250 mL flask at 220 rpm for 120 h at 30 °C after inoculating 10 mL seed culture. 0.1% (w/v) soybean oil was fed at the beginning of cultivation in the experimental group. Here, CsA was pre-dissolved in 95% ethanol solution and added into the cultivation medium at 24 h with an initial concentration of 600 mg/L (499.2 μmol/L).

### Analytical methods

The biomass yield was determined by dry cell weight (DCW). For the determination of the concentration of CsA and [(4′-OH)MeLeu]^4^-CsA, 5 mL culture fluid was immediately mixed with 5 mL 95% ethanol and shaken intermittently for 1 h. After centrifugation, the supernatant was subjected to HPLC (Agilent 1200, USA) equipped with an Eclipse XDB-C18 column (5 μm; 150 mm × 4.6 mm; Agilent Technologies) and a UV detector at 210 nm. The mobile phase was acetonitrile-0.1% phosphoric acid water solution (70:30, v/v) with a flow rate of 1.0 mL/min, and the column temperature was 60 °C.

The specific production rate (μp) is defined as μp = dp/dt/M, and the specific consumption rate is defined as μc = dc/dt/M, where p is the [(4′-OH)MeLeu]^4^-CsA production and c is the residual CsA in the fermentation broth, t is fermentation time and M is the dry weight of mycelium. dp/dt and dc/dt was calculated by the slope calculating plug-in “Tangent.opk” in Origin 8.1 (OriginLab, USA).

TMC represents the total molar concentration of the CsA and [(4′-OH)MeLeu]^4^-CsA in the fermentation broth, namely, TMC = C(CsA) + C(CsA − OH).

### Protein extraction and proteomics analysis

Protein extraction for 2-DE was carried out according to the previous work [38], and the experimental details were present in the Additional file 1 (Protein extraction and proteomics analysis). The protein concentration was measured by Bradford method [39]. 2-DE was performed at least in three biological replications for both control and soybean oil conditions. Isoelectric focusing was implemented using a Multiphor II electrophoresis system (Amersham Pharmacia Biotech, Uppsala, Sweden) at 20 °C for a total of 71,000 V h under 20 °C (S1: 0–500 V, 500 V h; S2: 500 V, 2500 V h; S3: 500–3500 V, 10,000 V h; S4: 3500 V, 50,000 V h; S5: 3500–500 V, 8000 V h). For each replicate, 0.8 mg protein was loaded onto a 17 cm immobilized pH gradient (IPG) strip (pH 4–7; Bio-Rad Laboratories, USA) mixed with 170 μL rehydration buffer (8 M urea, 2 M thiourea, 0.5% (w/v) CHAPS, 1% (w/v) DTT, 0.52% (w/v) Pharmalyte, and 0.002% (w/v) bromphenol blue). Prior to the second dimensional electrophoresis, IPG strips were equilibrated in two stages: reduction with DTT, then carboxymethylation with iodoacetamide [40]. The proteins in IPG strips were further separated using 12% sodium dodecyl sulphate–polyacrylamide gels (26 × 20 cm; Ettan DALT Twelve system with a programmable power controller) by Bio-Rad Protean II Xi system (Bio-Rad Laboratories, USA).

The protein staining was followed by Bradford method. The staining solution was composed of Coomassie brilliant blue R250 (0.25%), methanol (100 mL), milli-Q water (45 mL) and acetate (45 mL). The destaining solution was composed of ethanol (50 mL), acetate (100 mL) and milli-Q water (850 mL).

The staining gels were scanned at 300 dpi resolution by Umax Powerlook 2100XL Flatbed Scanner (UMAX Technologies Inc., Dallas, TX, USA) [41]. Subsequently, the image was analyzed with the Bio-Rad PDQuest Basic 2-D image processing software (version 8.0.1). By using PDQuest software, the average ratios and t test values for each spot and a value below 0.05 for t test was regarded as being significant. Protein spots with an average abundance change of greater than 1.5-fold and present in all biological replicates were subjected to MS analysis.

Protein spots were detained and digested as previously described [41]. More details were present in Additional file 1 (Protein extraction and proteomics analysis). The digested peptides were analyzed using a 4700 Proteomics Analyzer (Applied Biosystems, USA). The instrument was performed at a maximum accelerating potential of 20 kV and an *m*/*z* range from 700 to 4000. Six standards (Applied Biosystems, USA) were used as the internals to calibrate each spectrum to a mass accuracy within 0.1 Da. Protein candidate spots were analyzed using MALDI-TOF/TOF–MS in positive ion mode. Because the database of *N. dietziae* was uncompleted, proteins were identified by automated peptide mass fingerprinting using the Global Proteome Server Explorer software 3.0 (Applied Biosystems, USA) against a self-built protein sequence database of *N. candida*, *N. coxensis* DSM 45129 and *Nonomuraea* sp. SBT364 and some cyclosporine-specific P450 hydroxylases. For the algorithm, parameter settings were described by Wang et al. [42]. All the proteins identified were presented by MASCOT report protein scores for MS or total ion scores for MS/MS with greater than 95% confidence intervals.

### Sample preparation of intracellular metabolites for GC–MS

The experimental data was obtained from four replicates of each treatment. The samples of mycelium at 48, 72, 96 and 120 h were harvested for quenching and extraction of intracellular metabolites at a low temperature. Subsequently, the extracts were derived with a two-step method. The methods of sample preparation for GC–MS had been previously described [43] in the Additional file 1 (sample preparation of intracellular metabolites).

### Data acquisition and processing of GC–MS

GC–MS was performed by Agilent 6890N-5975C MSD system (Agilent Technologies, USA) equipped with a DB-5MS capillary column (30 m × 0.25 mm, 0.25 μm film thickness; Agilent Technologies, USA) and an autosampler. Parameter settings of GC–MS system were consistent with Wang’ work [44]. Metabolomic data was processed with the Agilent MSD ChemStation (Agilent Technologies, USA) for spectrum deconvolution, denoising, retention time aligning, peak area integration, compound identification combined with NIST mass spectrum database (http://webbook.nist.gov/chemistry/) [44].

### Gene expression determination by quantitative real time RT-PCR (qRT-PCR)

Real-time PCR was performed to detect the relative transcriptional expression levels of cytochrome P450 hydroxylase genes of *N. dietziae*. The samples were harvested at 36 and 48 h from the broth cultivation. Total RNA was extracted with RNA prep pure Cell/Bacteria Kit (TIANGEN, Beijing, China) according to the manufacturer’s protocol. The optical density at 260 and 280 nm was measured to determine the quantity and purity of RNA. cDNA was obtained by reverse transcription with total RNA as template using PrimeScript™ RT reagent Kit (Takara, Dalian, China) under instructions. On the basis of the CYP gene sequences of *N. dietziae*, the primer pairs were designed and listed in Additional file 1: Table S1, and the gene 16S *rDNA* was used as the internal control. Then, qRT-PCR analysis was operated in a 7500 Real-Time PCR Systems (Applied Biosystems, USA) with the TransStart Top Green qPCR SuperMix (TransGen Biotech, China) according to the manufacturer’s protocol by 2^−ΔΔCt^ method [45]. Three biological repetitions and two technical repetitions were implemented for each target gene.

## Additional file

**Additional file 1: Figure S1.** HPLC analysis of CsA and [(4’-OH)MeLeu]^4^-CsA. **Figure S2.** NMR analysis of CsA and [(4’-OH)MeLeu]^4^-CsA. **Figure S3.** HRMS analysis of CsA and [(4’-OH)MeLeu]^4^-CsA. **Figure S4.** 2DE-based proteomic profiles of *N. dietziae*. Proteins are extracted at different growth phases and media. Arrows point to the significantly differential proteins under MO condition and their characteristics are shown in Table 1. **Table S1.** Primers for qRT-PCR of the CYPs.

## Abbreviations

CsA: cyclosporin A
HIV: human immunodeficiency virus
ISP2 medium: yeast extract–malt extract agar
DCW: dry cell weight
2-DE: two-dimensional electrophoresis
GC–MS: gas chromatography–mass spectrometry
CYP: cytochrome P450 hydroxylase
EMP: Embden–Meyerh pathway
TCA: tricarboxylic acid
PPP: pentose phosphate pathway
ED pathway: Entner–Doudoroff pathway
G6PD: glucose-6-phosphate dehydrogenase
Edd: phosphogluconate dehydratase
PFK: 6-phosphofructokinase
ENO: enolase
PK: pyruvate kinase
DLD: dihydrolipoamide dehydrogenase
PDH: pyruvate dehydrogenase
CS: citrate synthase
KorA: 2-oxoglutarate ferredoxin oxidoreductase
SucA: alpha-ketoglutarate decarboxylase
sucD: succinyl-CoA synthetase alpha subunit
ASL: argininosuccinate lyase
NDH: NADH dehydrogenase
ALD: alanine dehydrogenase
LeuA: 2-isopropylmalate synthase
GLT: glutamate synthase
UROC1: urocanate hydratase
HAL: histidine ammonia-lyase
KDPG: 2-keto-3-deoxy-6-phosphogluconate
PBS: phosphate buffered solution
PMSF: phenylmethanesulfonyl fluoride
DTT: dithiothreitol
IPG: immobilized pH gradient
CHAPS: 3-[(3-cholamidopropyl) dimethylammonio]-1-propanesulfonate
