# Supplementary material for: Integrating multi-omics analyses of Nonomuraea dietziae to reveal the role of soybean oil in [(4′-OH)MeLeu]4-CsA overproduction
Source: Microb Cell Fact. 2017 Jul 14;16:120. doi: 10.1186/s12934-017-0739-0 (PMC5512743; doi:10.1186/s12934-017-0739-0)
Supplement: Supplementary file 1 — Additional file 1: Figure S1. HPLC analysis of CsA and [(4’-OH)MeLeu]4-CsA. Figure S2. NMR analysis of CsA and [(4’-OH)MeLeu]4-CsA. Figure S3. HRMS analysis of CsA and [(4’-OH)MeLeu]4-CsA. Figure S4. 2DE-based proteomic profiles of N. dietziae. Proteins are extracted at different growth phases and media. Arrows point to the significantly differential proteins under MO condition and their characteristics are shown in Table 1. Table S1. Primers for qRT-PCR of the CYPs. [file 12934_2017_739_MOESM1_ESM.docx]

**Supplementary Material**

**Integrating Mutli-omics Analyses of *Nonomuraea dietziae* to Reveal the Role of Soybean Oil in [(4’-OH)MeLeu]^4^-CsA Overproduction**

Huanhuan Liu^1,2^, Di Huang^3,4^, Lina Jin^1,2^, Cheng Wang^1,2^, Shaoxiong Liang^1,2^, Jianping Wen^1,2 *^

^1^Key Laboratory of System Bioengineering (Tianjin University), Ministry of Education, Tianjin, 300072, People’s Republic of China

^2^SynBio Research Platform, Collaborative Innovation Center of Chemical Science and Engineering (Tianjin), School of Chemical Engineering and Technology, Tianjin University, Tianjin, 300072, People’s Republic of China

^3^TEDA Institute of Biological Sciences and Biotechnology, Nankai University, TEDA, Tianjin 300457, People’s Republic of China

^4^SynBio Research Platform, Collaborative Innovation Center of Chemical Science and

Engineering (Tianjin), Nankai University, Tianjin, 300074, People’s Republic of China

Huanhuan Liu, Di Huang and Lina Jin contributed equally to this work.

*Correspondence to: Jianping Wen

Telephone & Fax: +86-022-27892061;

E-mail: jpwen@tju.edu.cn

**CONTENTS**

[1. HPLC analytical method 1](#_Toc485932340)

[Figure S1 HPLC analysis of CsA and [(4’-OH)MeLeu]^4^-CsA 2](#_Toc485932341)

[2. NMR spectroscopy 3](#_Toc485932342)

[Figure S2 NMR analysis of CsA and [(4’-OH)MeLeu]^4^-CsA 4](#_Toc485932343)

[3. Mass spectrometry 5](#_Toc485932344)

[Figure S3 HRMS analysis of CsA and [(4’-OH)MeLeu]^4^-CsA 6](#_Toc485932345)

[4. Protein extraction and proteomics analysis 7](#_Toc485932346)

[Figure S4 2DE-based proteomic profiles of *N. dietziae*. 9](#_Toc485932347)

[6. Sample preparation of intracellular metabolites for GC-MS 12](#_Toc485932348)

[7. Data processing and statistical analysis of metabolomics 13](#_Toc485932349)

8. [Table S1 Primers for qRT-PCR of the CYPs 14](#_Toc485932350)

1. **HPLC analytical method**

For the determination of the concentration of CsA and [(4’-OH)MeLeu]^4^-CsA, 5 mL culture fluid was immediately mixed with 5 mL 95 % ethanol and shaken intermittently for one hour. After centrifugation, the supernatant was subjected to HPLC (Agilent 1200, USA) equipped with an Eclipse XDB-C18 column (5 μm; 150 mm×4.6 mm; Agilent Technologies) and a UV detector at 210 nm. The mobile phase was acetonitrile-0.1 % phosphoric acid water solution (70:30, v/v) with a flow rate of 1.0 mL/min, and the column temperature was 60 °C.

The retention time of CsA is about 12.7 min and 20.5 min of [(4’-OH)MeLeu]^4^-CsA as shown in Fig. S1.


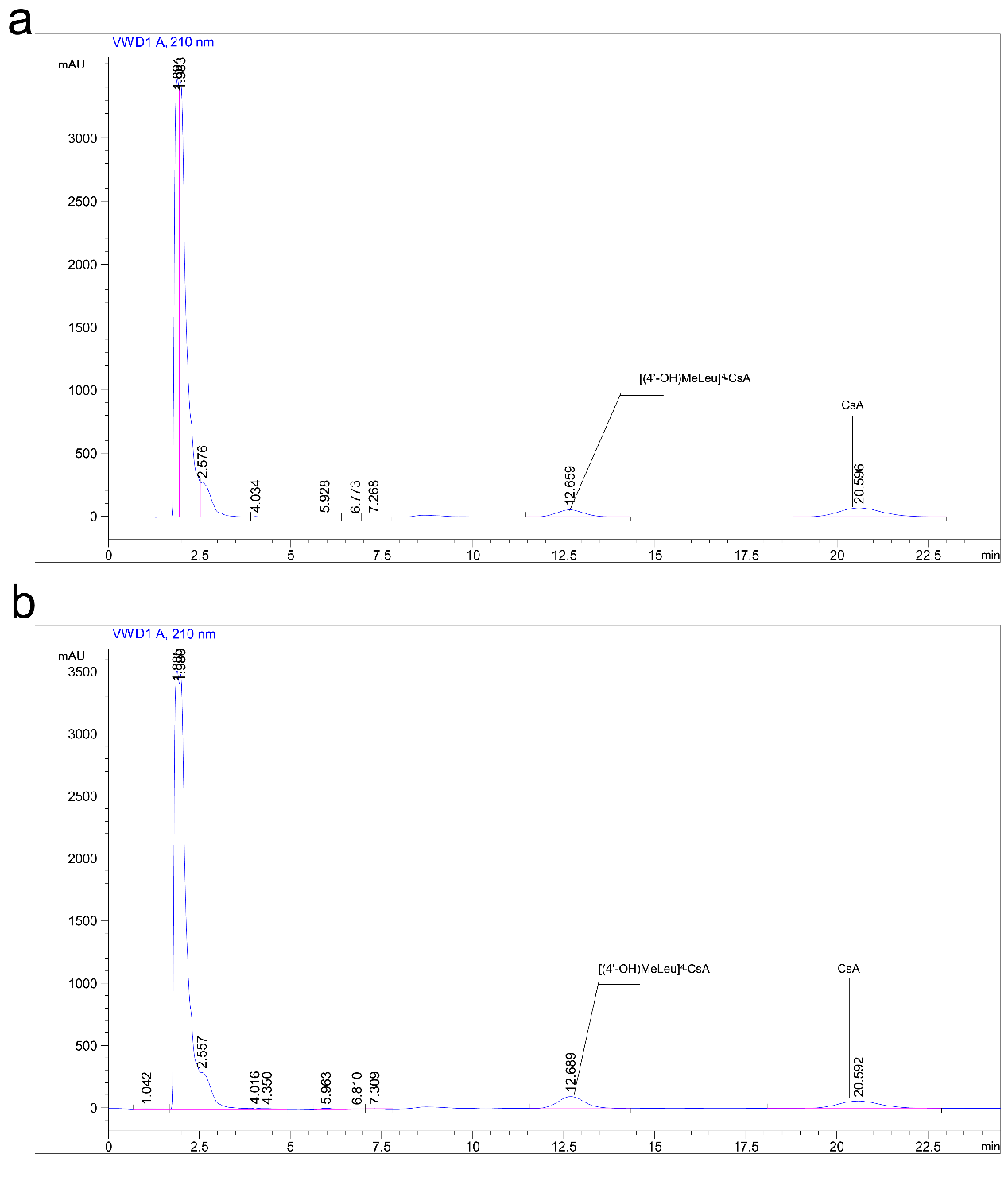


**Figure S1** HPLC analysis of CsA and [(4’-OH)MeLeu]^4^-CsA in the fermentation broth. a) The control condition; b) The soybean oil condition

1. **NMR spectroscopy**

Prep-HPLC purification was performed for NMR and HRMS analysis.

1H-NMR analysis was carried out with a Bruker Avance 600 MHz spectrometer in methanol-D4 at 600.17 MHz at the School of Pharmaceutical Science and Technology of Tianjin University, PRC.

The results of NMR spectroscopy analysis for CsA and [(4’-OH)MeLeu]^4^-CsA were shown in Fig. S2. The NMR spectrum of CsA is consistent with the previous work (Xu et al, 2012).

**Reference**

Wu X, Stockdill J L, Park P K, et al. Expanding the Limits of Isonitrile-Mediated Amidations: On the Remarkable Stereosubtleties of Macrolactam Formation from Synthetic Seco-Cyclosporins[J]. Journal of the American Chemical Society, 2012, 134(4):2378-84.

**
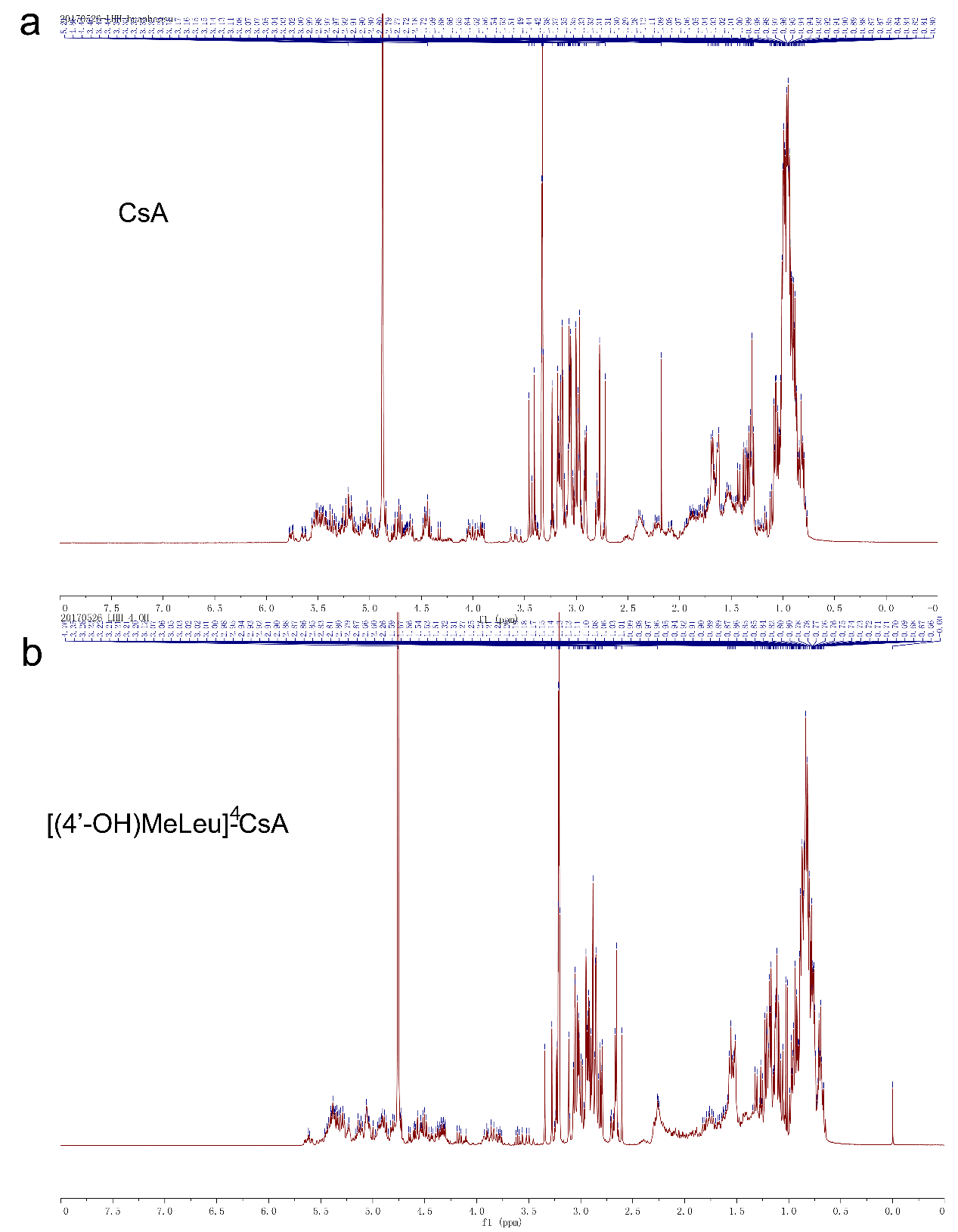
**

Figure S2 NMR analysis of CsA and [(4’-OH)MeLeu]^4^-CsA

1. **Mass spectrometry**

To confirm the integrity of the compounds, purified products were analyzed by high resolution mass spectra (HRMS, m/z) with a Bruker MicroTOF spectrometer using positive (ESI+) ionization using the following settings:

Source Type, ESI; Set Nebulizer, 0.4 Bar; Ion Polarity, Positive; Focus, Active; Set Capillary, 4500 V; Set Dry Heater, 180 °C; Scan Begin, 500 m/z; Set End Plate Offset,-500 V; Set Dry Gas 4.0 l/min; Scan End, 1500 m/z; Set Collision Cell RF; 700.0 Vpp Set Divert Valve, Waste.

The results of mass spectrometry analysis for CsA and [(4’-OH)MeLeu]^4^-CsA were shown in Fig. S3. HRMS (ESI+) m/z calculated for [M+Na]+ (C_62_H_111_N_11_O_12_Na, CsA-Na): 1224.8311, found:1224.8386; [M+Na]+ (C_62_H_111_N_11_O_13_Na, [(4’-OH)MeLeu]^4^-CsA-Na): 1240.8301, found:1240.8312.


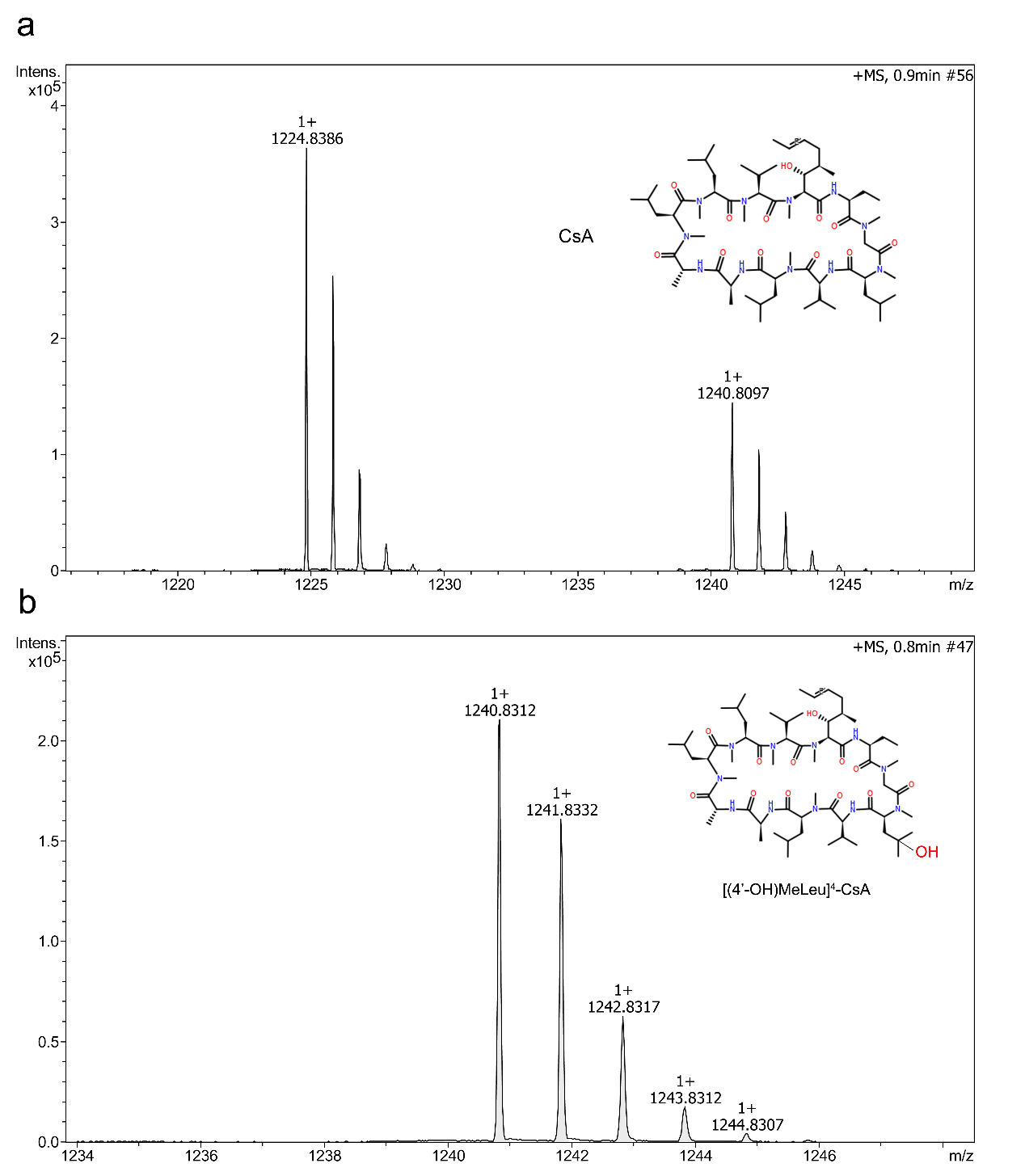


Figure S3 HRMS analysis of CsA and [(4’-OH)MeLeu]^4^-CsA

1. **Protein extraction and proteomics analysis**

Protein extraction for 2-DE was carried out according to the previous work (Martinez-Moya et al. 2015). The experimental detail was present in the supporting information (S1). Briefly, culture samples were centrifuged at 4 °C, 12,000 × g for 10 min and washed thoroughly with precooled phosphate buffered solution (PBS). The precipitated cells were grinded in liquid nitrogen for cell disruption and then re-dissolved in PBS buffer containing 1 mM phenylmethanesulfonyl fluoride (PMSF) and 1 % dithiothreitol (DTT). Cells were further broken by mild sonication using a UP200S Cell sonicator for 5 cycles of 1 second pulse with 2 seconds gap on ice. After centrifugation at 14,000 rpm for 30 min at 4 °C, proteins released in the supernatant were harvested. Subsequently, a four-fold volume of 10 % precooled trichloroacetic acid-acetone was added and precipitated overnight at -20 °C. The protein pellet was then washed twice with pre-chilled 90 % acetone, and centrifuged at 12,000 × g for 30 min at 4 °C. After air-drying the protein pellet, proteins were dissolved in lysis buffer containing 8 M urea, 2 M thiourea, 2 % (w/v) 3-[(3-cholamidopropyl) dimethylammonio]-1-propanesulfonate (CHAPS), 2 % (w/v) ampholyte, 1 % DTT and 1 mM PMSF. The protein concentration was measured by Bradford method using bovine serum albumin as the standard.

2-DE was performed at least in three replications for both control and soybean oil conditions to minimize errors. Isoelectric focusing was performed using a Multiphor II electrophoresis system (Amersham Pharmacia Biotech, Uppsala, Sweden) at 20 °C for a total of 71,000 Vh under 20 °C (S1:0-500 V, 500 V h; S2: 500 V, 2500 V h; S3: 500-3500 V, 10,000 V h; S4: 3500 V, 50,000 V h; S5: 3500-500 V, 8000 V h). For each replicate, 0.8 mg protein was loaded onto a 17 cm immobilized pH gradient (IPG) strip (pH 4–7; Bio-Rad Laboratories, USA) mixed with 170 μL rehydration buffer (8 M urea, 2 M thiourea, 0.5 % (w/v) CHAPS, 1 % (w/v) DTT, 0.52 % (w/v) Pharmalyte, and 0.002 % (w/v) bromphenol blue). Prior to the second dimensional electrophoresis, IPG strips were equilibrated in two stages: reduction with DTT, then carboxymethylation with iodoacetamide (Wang et al. 2014). The proteins in IPG strips were further separated using 12 % sodium dodecyl sulphate-polyacrylamide gels (26×20 cm; Ettan DALT Twelve system with a programmable power controller) by Bio-Rad Protean II Xi system (Bio-Rad Laboratories, Hercules, CA, USA).

The gels stained with staining solution were scanned at 300 dpi resolution by Umax Powerlook 2100XL Flatbed Scanner (UMAX Technologies Inc., Dallas, TX, USA) (Zhao et al. 2012). Subsequently, the image was analyzed with the Bio-Rad PDQuest Basic 2-D image processing software (version 8.0.1). Protein spots on gels were detected, background subtracted, matched, and quantified. By using PDQuest software, the average ratios and t-test values for each spot and a value below 0.05 for t-test was regarded as significant. Protein spots with an average abundance change of at least 1.5-fold and present in all biological replicates were subjected to MS analysis.

The 2DE-based proteomic profiles of *Nonomuraea dietziae* under the control and soybean oil conditions were presented in Fig. S4.


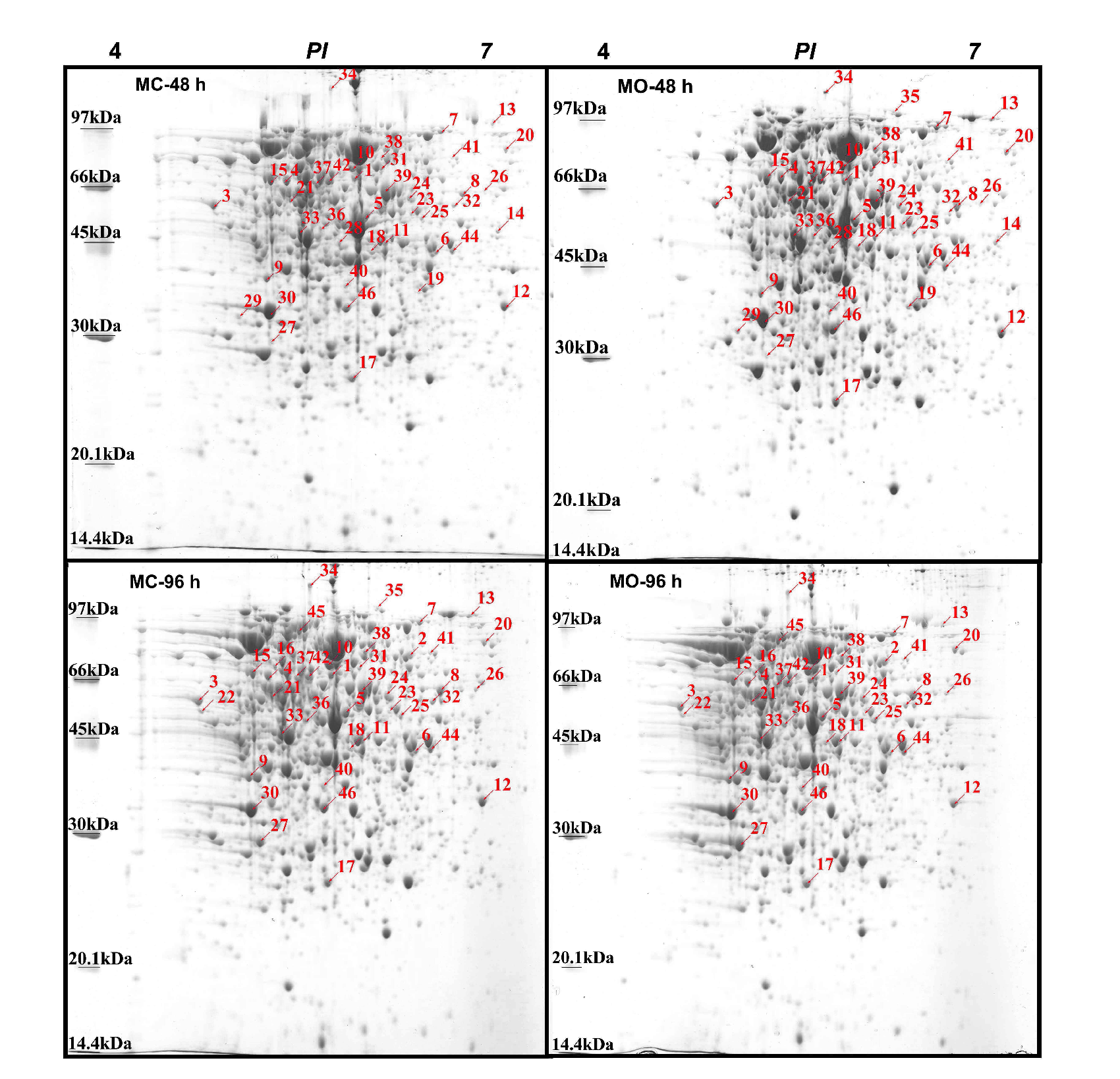


Figure S4 2DE-based proteomic profiles of *N. dietziae* under the control and soybean oil conditions. MC, the control media; MO, the soybean oil media.

1. **Identification of the protein spots**

Protein spots were detained and digested as previously described (Zhao et al. 2012). More details were present in supporting information (S1). Briefly, protein spots were cut from gels, washed with distilled water, destained with aqueous 50 % acetonitrile (pH 8.0) containing 25 mM ammonium bicarbonate, followed by distilled water. The destained gel fragments were dehydrated by immersion in 30 μL 100 % acetonitrile for 5 min and dried at room temperature. The dried gel fragments were digested with 8 μL trypsin buffer (0.1 mg/mL in 25 mM ammonium bicarbonate) at 37 °C for 16 h. After centrifugation, the supernatant containing peptides (0.3 μL) was mixed with an equal volume of a saturated solution of α-cyano-4-hydroxycinnamic acid in 50 % acetonitrile containing 0.1 % trifluoroacetic acid. Then the samples were spotted onto the target wells of sample plates, dried at room temperature, and analyzed using a 4700 Proteomics Analyzer (Applied Biosystems). The instrument was performed at a maximum accelerating potential of 20 kV and an m/z range from 700 to 4000. Six standards (Applied Biosystems) were used as the internals to calibrate each spectrum to a mass accuracy within 0.1 Da. Protein candidate spots were analyzed using MALDI-TOF/TOF-MS in positive ion mode. Because the database of *N. dietziae* was uncompleted, proteins were identified by automated peptide mass fingerprinting using the Global Proteome Server Explorer software 3.0 (Applied Biosystems) against a self-built protein sequence database of *N. candida*, *N. coxensis* DSM 45129 and *Nonomuraea* sp. SBT364 and some cyclosporine-specific P450 hydroxylases. For the algorithm, trypsin was used as the proteolytic enzyme, a maximum of a missed cleavage site was accepted. Besides, carbamidomethyl of cysteine and oxidization methionine was a fixed and variable modification, respectively. Peptide and fragment mass tolerances were set to ±0.1 Da and ±0.3 Da, respectively. All the proteins identified were presented by MASCOT report protein scores for MS or total ion scores for MS/MS with greater than 95 % confidence intervals.

**Reference**

Martinez-Moya P, Niehaus K, Alcaino J, Baeza M, Cifuentes V. 2015. Proteomic and metabolomic analysis of the carotenogenic yeast Xanthophyllomyces dendrorhous using different carbon sources. BMC Genomics 16:289.

Wang C, Chen J, Hu WJ, Liu JY, Zheng HL, Zhao F. 2014. Comparative proteomics reveal the impact of OmcA/MtrC deletion on Shewanella oneidensis MR-1 in response to hexavalent chromium exposure. Appl Microbiol Biotechnol 98(23):9735-9747.

Zhao G, Hou L, Yao Y, Wang C, Cao X. 2012. Comparative proteome analysis of Aspergillus oryzae 3.042 and *A. oryzae* 100–8 strains: towards the production of different soy sauce flavors. J. Proteomics 75(13):3914-3924.

**6. Sample preparation of intracellular metabolites for GC-MS**

The experimental data was obtained from four replicates of each treatment. Here, samples quenching and extraction of intracellular metabolites were performed at a low temperature. At each time point (48, 72, 96 and 120 h), 10 mL culture broth was quenched with a four-fold volume of pre-chilled methanol-water solution (60 %) in a 50 mL tube, then quickly mixed with a vortex mixer (HYQ-3110; Crystal Technology, USA) for 2-5 s and stored in the prepared ice box about 5 min to terminate the intercellular metabolic reactions. Subsequently, the mixture was centrifuged at 8000 × g for 15 min at -20 °C and washed three times by 10 mL 4 °C PBS. The resulting cell was re-suspended by -40 °C 50 % (v/v) methanol-water solution in a 1.5 mL Eppendorf tube. Then a freeze-thaw operation was carried out for three times in liquid nitrogen. After centrifugation, the supernatant was collected and blended with 50 µL succinic acid-2,2,3,3-d4 (0.2 mg/mL) as the internal standard, and then freeze-dried for 12 h (Alpha 1-2LD PLUS, Christ, Germany).

Subsequently, the samples were derived with a two-step method.

Derivatization was used for lowering the polarity of the compounds and gasification temperature to expand the spectrum of GC-MS. Here, we adopted the silane derivatization approach. In detail, 50 µL of 20 mg/mL methoxylamine hydrochloride - pyridine solution was added into the sample, then kept at 40 °C for 90 min. Subsequently, 80 µL N-methyl-N-(trimethylsilyl) trifluoroacetamide was added and mixed, later kept at 40 °C for 30 min.

**7. Data processing and statistical analysis of** **metabolomics**

GC-MS was performed by Agilent 6890N-5975C MSD system (Agilent Technologies) equipped with a DB-5MS capillary column (30 m×0.25 mm, 0.25 μm film thickness; Agilent Technologies) and an Agilent 7683B autosampler. GC-MS relevant parameter settings of GC-MS system were consistent with Wang *et al*. (Wang et al. 2015).

Metabolomic data was processed with the Agilent MSD ChemStation (Agilent Technologies Inc. USA) for MS spectrum deconvolution, denoising, retention time aligning, peak area integration and compound identification combined with NIST mass spectrum database (http://webbook.nist.gov/chemistry/). The detected compounds that could be found in the KEGG metabolite database (http://www.genome.jp/kegg/) were acceptable for the further analysis. Relative abundance of each metabolite was calculated by peak area normalization with internal standard and cell biomass.

**8. Table S1 Primers for qRT-PCR of the CYPs in this study**

| Sb1 | 5' TGGCGAGGCGTGGTGGGTGT 3' |
| --- | --- |
|  | 5' GGGCAGCGGCGTGTTCGTTG 3' |
| Sb2 | 5' CCGCAAGGAGTCCGAACCC 3' |
|  | 5' CCGTCGGCGATGGTGAAGT 3' |
| Sb3-1 | 5' TAGGGCCGATTTCCGCAGGTG 3' |
|  | 5' TGCCGTTGCCGATGAGGTTGA 3' |
| Sb3-2 | 5' GCTGGTCAACCCCACCTTCGT 3' |
|  | 5' GCAGGCTGAGCGTGGAGATGA 3' |
| Sb4 | 5' GCGTTCCGCCTTCTGTGCCA 3' |
|  | 5' CGACCGTGCCCTTGGGAATC 3' |
| Sb6 | 5' GCCGACGAGTTGTTCGACGATC 3' |
|  | 5' CGTCCATGCGGAGGAAGAAGC 3' |
| Sb7 | 5' TCGGAGGCTTCGTTCACCCC 3' |
|  | 5' CGGATCGGCCAGTGCAGGTT 3' |
| Sb8 | 5' CCGCCACTCCGACCTGTTCT 3' |
|  | 5' ACCTTCTGGTGTGCTCGGGC 3' |
| Sb9 | 5' CGAACCCACAGGCTTTCTACCGC 3' |
|  | 5' CCCTTGACCAGTTCCGACAGCG 3' |
| Sb10 | 5' CTCACCGAGCTGGAACGGATGT 3’ |
|  | 5' GCTCCGAACCCGAAGGAAAGG 3' |
| Sb11 | 5' CGGTGCGGTGGGTGTGGTCTAC 3' |
|  | 5' TACGGGATGTCGTCGGGGTCG 3' |
| Sb12 | 5' CGAGGCGACCGTGAACGTC 3' |
|  | 5' CCGAAGGAGATGTGCGGGTTGT 3 |
| Sb13 | 5' CTCACCAACATCGGGGTTCTGC 3' |
|  | 5' CGGTGTGGGTGATGCTCAGGTAG 3' |
| Sb15 | 5' CTTCCCTTCTACCGTGCCG 3' |
|  | 5' CCAGCGAGGCGGTGTATCT 3' |
| Sb16 | 5' TGACTCCCAGCCCCTCACCC 3' |
|  | 5' GCGACGGAACCGCCTGTGAT 3' |
| Sb17 | 5' AGCCAGGAAGAGCAGGACAAGC 3' |
|  | 5' CCTACGGCACTCCCAGCAACT 3' |
| Sb20 | 5' GTGGGCGACACCCGTTTCAGT 3' |
|  | 5' GCCGTCTGAGCCTGGTGTGC 3' |
| Sb21 | 5' TGTCAAGACCGTGCTGGGC 3' |
|  | 5' GGACGCATCTCCATGACGC 3' |
| Sb22 | 5' GTCTACCGGATGGAGATCAAGCAG 3' |
|  | 5' CGAATCGCTGGGCGTTGTC 3' |
| Sb23 | 5' GCTGTCGGCCAACTGGGACC 3' |
|  | 5' AGCCGCAGGGAGGGGATACG 3' |
| Sb24 | 5' ACGGGCGGATGACCGAAGAG 3' |
|  | 5' TTGGCGTCGTAGCGCAGGCT 3' |
